# Bimetallic *bis*-Aroyldihydrazone-Isatin Complexes of High O=V(IV) and Low Cu(II) Valent Ions as Effective Biological Reagents for Antimicrobial and Anticancer Assays

**DOI:** 10.3390/molecules29020414

**Published:** 2024-01-15

**Authors:** Ahmed Khalil, Mohamed Shaker S. Adam

**Affiliations:** 1 Department of Chemistry, College of Science, King Faisal University, P.O. Box 400, Al-Ahsa 31982, Saudi Arabia; madam@kfu.edu.sa; 2 Chemistry Department, Faculty of Science, Zagazig University, Zagazig 44519, Egypt; 3 Department of Chemistry, Faculty of Science, Sohag University, Sohag 82534, Egypt

**Keywords:** dihydrazone diisatin, oxovanadium (IV), dinuclear VO^2+^ and Cu^2+^ complexes, antimicrobial assay, antiproliferative assay, *ct*DNA reactivity

## Abstract

Due to the versatile bioreactivity of aroyldihydrazone complexes as cost-effective alternatives with different transition metals, two novel bimetallic homo-complexes (VOLph and CuLph) were prepared via the coordination of a terephthalic dihydrazone diisatin ligand (H_2_Lph) with VO^2+^ and Cu^2+^ ions, respectively. The structure elucidation was confirmed by alternative spectral methods. Biologically, the H_2_Lph ligand and its MLph complexes (M^2+^ = VO^2+^ or Cu^2+^) were investigated as antimicrobial and anticancer agents. Their biochemical activities towards *ct*DNA (calf thymus DNA) were estimated using measurable titration viscometrically and spectrophotometrically, as well as the gel electrophoresis technique. The growth inhibition of both VOLph and CuLph complexes against microbial and cancer cells was measured, and the inhibition action, MIC, and IC_50_ were compared to the inhibition action of the free H_2_Lph ligand. Both VOLph and CuLph showed remarkable interactive binding with *ct*DNA compared to the free ligand H_2_Lph, based on *K*_b_ = 16.31, 16.04 and 12.41 × 10^7^ mol^−1^ dm^3^ and $\Delta G_{b}^{\neq }$ = 47.11, −46.89, and −44.05 kJ mol^−1^ for VOLph, CuLph, and H_2_Lph, respectively, due to the central metal ion (V^IV^O and Cu^II^ ions). VOLph (with a higher oxidation state of the V^4+^ ion and oxo-ligand) exhibited enhanced interaction with the *ct*DNA molecule compared to CuLph, demonstrating the role and type of the central metal ion within the performed electronegative and electrophilic characters.

## 1. Introduction

Intensive alternative synthetic strategies of aromatic hydrazones with different heteroatomic functional groups have been established for a wide array of applications in the pharmaceutical and medicinal fields [1]. Aryl/aroyl hydrazones containing a >C=N–NH–C=O linkage have been synthesized using facile and low-cost procedures [2].

The indispensable steric and electronic features of aryl/aroyl hydrazones control their enforced ability to form highly stable metallo-organic complexes with different geometrical structures with transition metals of alternative positive oxidation states [2]. The coordination attitude of such aryl/aroyl hydrazone-based pincer chelating agents towards transition metal ions was monitored via the substitution of the *N*,*O*-donor atoms of the σ-/π-donating-accepting electronic force and the bite angles of the complexation with their remarkable steric effect [2]. The chemical characteristics of aryl/aroyl hydrazones and their metal organic–base complexes are of interest in various applicable areas, such as chemosensors [3], electroluminescent fluorescence agents [4], photovoltaic nonlinear optical precursors [5] and medicinal reagents [1,2], encouraging inorganic chemists to design new derivatives of hydrazone-based metal complexes.

The addition of molecular species, such as metal-based complexes of specific metal–organic frameworks, has been used to develop new drug candidates for pharmacological and medicinal applications [6]. Metal-based organic complexes have been widely used in medicinal fields, i.e., cisplatin, carboplatin, and oxaliplatin, as the most famous and international anticancer drugs with considerable toxicity, drug resistance, ototoxicity, nephrotoxicity, and renal impairment [6]. Therefore, many large metal–organic framework complexes consisting of positively charged M^n+^ ions have been constructed, providing enhanced medicinal applicability and fewer biological side effects [7]. In addition, derivatives of chelating metal–organic hydrazone complexes were designed for their effectiveness as anticancer candidates and antimicrobial reagents, considering the theory of Tweedy’s chelating effect [8] to distinguish their improved Lewis acidic characters and lipophilicity compared to their free aryl/arylhydrazone-based ligands. These features could be the reason for their penetration into the microbial and cancer cell walls and their interacting or distorting effect on cell DNA [9].

The improvement of toxicity and the harmless side effects of vanadium–organic complexes have motivated many researchers to design V^IV^O-, V^V^O- and VO_2_- and Cu(I/II)-coordination compounds of various backbone organo-ligands [10]. The significant antibacterial reactivity of newly constructed vanadium (III) complexes of hydrazido-hydrazone ligands was also reported [11]. Hydrazone derivatives of Schiff base chelates with VO^2+^, Zn^2+^, and Cu^2+^ have been employed against DNA to evaluate their interaction binding effect [12]. Back et al. [13] also reported *ct*-DNA and HAS interaction assays with newly prepared V^V^O-hydrazone complexes meditated by docking assessments. Desalegn et al. [14] investigated the antioxidant and antibacterial potential of two novel cobalt (II) and oxovanadium (IV) coordination compounds of a quinolone-substituted hydrazone ligand. The role of the coordinate hydrazone chelates, as a bioligand, was discovered via in vitro cytotoxicity examination for the dinuclear complexes of dioxo-vanadium (V) ions [15]. The biological importance of such hydrazone ligands in their coordinated forms and their relationship with vanadium and molybdenum ions, as pyridoxal derivatives, have also been examined and reported [16], referring to the functionalized role of V^4+^O and Mo^6+^O_2_ ions in hydrazone base complexes.

One of the most versatile transition elements is copper (as a very suitable and cheap element), with structural and chemical potential for various proposes [17]. Currently, many organic derivatives of copper (II) hydrazone-based organometallic reagents have been synthesized for many biological studies, such as Cu^2+^, Ni^2+,^ and Fe^3+^ complexes derived from the dioxanylidene hydrazinyl benzoic acid ligand, as highly reactive antimicrobial reagents [18]. Furthermore, Samy and Shebl reported the antitumor activity of newly designed *N*,*O*-hetero pincer chelating hydrazone complexes of copper(II), nickel(II), and cobalt(II) ions within docking studies against hepatocellular carcinoma [19]. Additionally, new chemotherapeutic metallo-organic chelates have been designed, such as the new Schiff base hydrazone complexes of Pd^2+^, Cu^2+,^ and Cd^2+^ ions [20] and of Co^2+^ and Cu^2+^ ions [21], and have been biologically screened as treatments for COVID-19. Many M^II^-hydrazone-based chelated coordination compounds have been employed in various biological assays [22] and in in vitro studies employing in silico methods [23]. The influence of the alternative central metal ion of different oxidation states was explored in new hydrazone-based complexes [24], which have been applied in various important biological metabolisms to address medicinal and pharmaceutical demand, e.g., SOD mimetic reactivity [25,26]. Significantly, metallo-triazine hydrazone pincer chelates have been constructed via the coordination between Cu^II^ and Zn^II^ ions concerning their behavior against the cytokine genes and peptide growth action in rabbits [27].

In this work, new homoleptic bimetallic diisatin-dihydrazone pincer chelates of vanadyl (IV) and copper (II) ions were constructed as effective biological reagents against some common microbial (fungi/bacteria) and human cancer cell lines. The interaction mode of the synthetic compounds was estimated using three strategies, namely gel electrophoresis, electronic spectroscopy, and viscosimetry, exhibiting the role of the positively charged M^n+^ ion in the progression of the interaction with *ct*DNA.

## 2. Results and Discussion

### 2.1. Preparation and Structural Elucidation

**Scheme 1:**
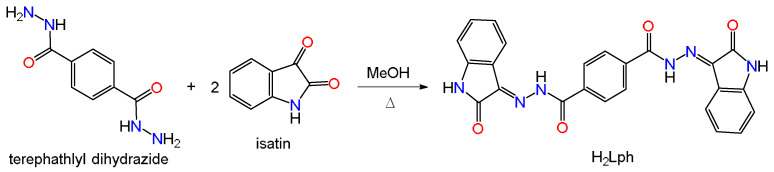
Condensiation reaction of terephathlyl dihydrazide with isatin, producing H_2_Lph as a chelating ligand.

**Scheme 2:**
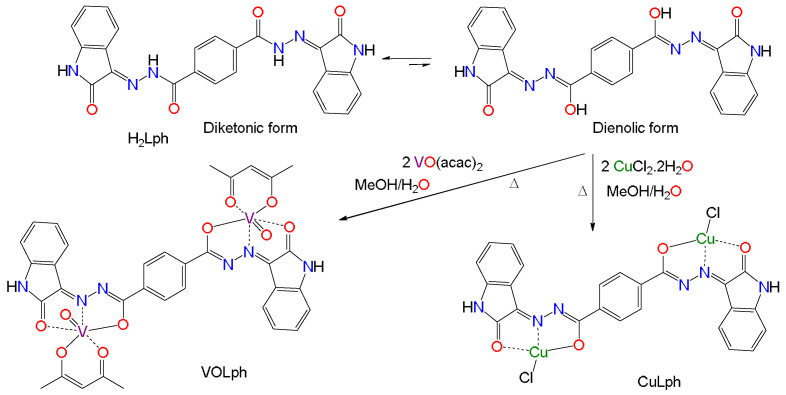
Complexing pathway of a VO^2+^ or Cu^2+^ ion with the H_2_Lhm ligand to prepare VOLph and CuLph, respectively, at 80 °C.

As reported for succinate and oxalate derivatives [28,29], H_2_Lph was prepared by adding 1 equivalent of terephthalic dihydrazide with 2 equivalents of isatin in methanol under the conditions of a condensation reaction to produce the target ligand of terephthalic dihydrazone diisatin (H_2_Lph) (Scheme 1). An aqueous solution of divalent metal ions, consisting of VO^2+^ or Cu^2+^ ions of oxovanadium acetylacetonate or copper chloride dihydrate, respectively, was coordinated with H_2_Lph to form two new VOLph and CuLph complexes, as bimetallic homoleptic chelates. H_2_Lph was coordinated with VO^2+^ or Cu^2+^ ions within the enolic structure at 1:2 equivalent ratios, as a *bis*-tridentate (*N*,*O*, and *O*-atoms) and monobasic pincer chelated ligand, to produce VOLph and CuLph with a 68% and 71% yield, respectively, as shown in Scheme 2. The chemical structures of H_2_Lph, VOLph, and CuLph were elucidated by means of classical spectroscopic analyses, such as EI-Mass, NMR, infrared, and UV/Vis spectra. Moreover, the current compounds were examined via elemental composition analyses (EA), magnetic feature analysis, and the conductance commentary, as presented in Table 1. Both MLph complexes (VOLph and CuLph) displayed a paramagnetic character, with an assigned magnetic moment of 1.98 and 2.13 B.M, respectively, attributed to one free (unpaired) electron in the valence shell with *d*^1^ and *d*^9^ electronic distribution for the V^4+^ ion with an oxo-ligand and a Cu^2+^ ion, respectively [30,31].

The carbon, hydrogen, and nitrogen percentages in the molecular structures of H_2_Lph, VOLph, and CuLph were estimated, as listed in Table 1. The main percentages of the ligand and its MLph complexes were adopted and compared with the expected values (with differentiation of less than 0.4%). In particular, the melting and/or decomposition points were determined to be 208 °C (for the free ligand) and >300 °C (for both complexes), respectively, demonstrating the high stability of MLph chelates (VOLph and CuLph) compared to H_2_Lph. The difference in stability was attributed to the coordination bonding and chelating action of H_2_Lph towards VO^2+^ and Cu^2+^ ions [32,33]. Accordingly, in DMF, the molar ratios of VO^2+^ or Cu^2+^ ions to H_2_Lph (the coordinated ligand) to form VOLph or CuLph chelates (the stoichiometry) was examined spectrophotometrically using the continuous variation method [34,35,36]. As shown in Figure S3, the results demonstrate that both VOLph and CuLph chelates were synthesized by means of the coordination of H_2_Lph with VO^2+^ and Cu^2+^ ions at 1:2 equivalent ratios, respectively (Scheme 2). As shown in Figure S4, the pH stability was also detected for DMF solutions of VOLph and CuLph in various pH media using standard universal buffer solutions [37]. VOLph and CuLph chelates exhibited a wide range of pH stability at 33.3–10.6. H_2_Lph, VOLph, and CuLph were highly soluble in dimethylsulfoxide (DMSO) and *N*,*N′*-dimethylformamide (DMF), while having less solubility in the polar solvents (e.g., methanol, acetonitrile, ethanol, and acetone) and low-polar organic solvents (e.g., dichloromethane, chloroform, and benzene). In addition, the conductivity results (Table 1) for the VOLph and CuLph complexes probably point to their non-polar features (no counter ions could be found) [38]. However, the conductivity data of the VOLph and CuLph complexes in DMF were slightly inferior compared to those for the 1:1-type electrolytes. Hence, a partial substitution of the chlorido and acetylacetonato ligands with the DMF molecule in VOLph and CuLph complexes could be strongly considered. The coordination behavior of the chloride ion in CuLph was detected with MS spectroscopy; however, it could be also distinguished experimentally through qualitative analysis with an aqueous solution of AgNO_3_ by mixing the AgNO_3_ solution with a solution of CuLph in MeOH/DMF. In particular, no precipitation was observed in the reaction mixture resulting from AgCl. Consequently, a Cl^−^ anion was found in the coordination sphere of the CuLph complex, not as a counter ion. The low-polar nature of CuLph, combined with the low conductivity measurements, supports the above results (Scheme 2).

#### 2.1.1. NMR Spectra of H_2_Lph Organo-Ligand

**Figure 1:**
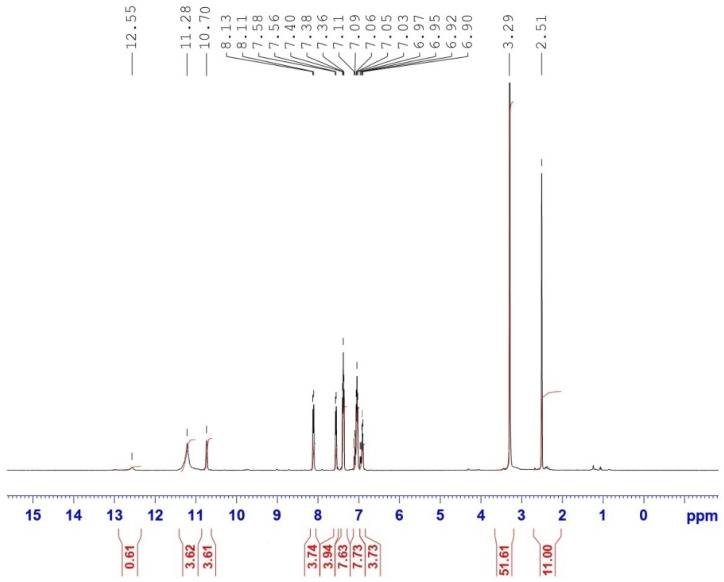
^1^H-NMR spectroscopy of the H_2_Lph ligand in DMSO-*d*_6_ at room temperature.

The ^1^H and ^13^C NMR spectra of H_2_Lph were examined in DMSO-*d*_6_ at an ambient temperature, and are illustrated in Figures S1 and S2, respectively. Notably, the two protons of the N–H bonds of dihydazone and isatin substituents in the ketonic structure of H_2_Lph were positioned as broad signals at 11.28 and 10.70 ppm, respectively [29,39]. Also, the residual proton absorption spectral signals for H_2_Lph, which characterized the aromatic rings, were in the range of 8.13–6.90 ppm (Figure 1). The ^1^HNMR spectrum, as given in Figure 1, exhibited some additional small signals other than those of the H_2_Lph sample, which belonged to the enolic tautomer. A spectral signal was present at 12.55 ppm, representing the N–H proton of the isatin moiety of the enolic tautomer. Moreover, new spectral signals were observed for the aryl rings in the enolic tautomer at 6.96 and 7.10 ppm, as shown in Figure 1.

Based on the ^13^CNMR spectrum, the carbon spectral signals for the two carbonyl (C=O) groups in the dihydrazone chain were found at 165.11 and 162.81 ppm (Figure S2) in the ketonic tautomer. The absorption signal of the >C=N– carbon was located at 150.02 ppm. The other spectral signals were related to the carbons of the aromatic rings of the terephthalate and isatin moieties [40].

#### 2.1.2. UV and Vis. Spectroscopy

**Figure 2:**
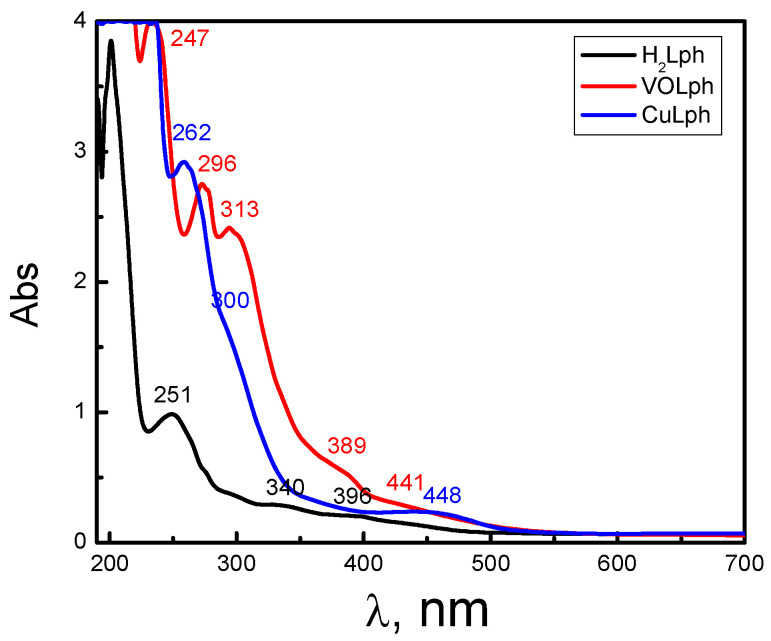
Scanning of the H_2_Lph, VOLph, and CuLph solutions at 25 °C (in DMF) with 1.0 × 10^−6^ mol dm^−3^ in UV-Vis regions for the electronic spectra.

At a 1.0 × 10^−6^ mol dm^−3^ concentration of the H_2_Lph ligand and its metal chelates VOLph and CuLph, the detectable molecular electronic transitions were characterized by λ_max_ (i.e., wavelengths for the optimized absorption) and the corresponding ε (the molecular absorptivity), which are shown in Figure 2 and listed in Table 1. Therefore, the possible electronic absorption of the π→π*/n→π* types were distinguished at 251 and 340 nm (for H_2_Lph), 296 and 313 nm (VOLph), and 262 and 300 nm (CuLph) in the ultraviolet area, respectively. Those modes of transition were observed for the transition of the π electrons and lone pairs of the >C=O, –NH, and >C=N– groups and the aryl ring π electron system to the highly energetic empty π* orbitals [41].

The matched absorption bands for the ligand charge transfer in the uncoordinated ligand molecule (LCT) and the charge transfer from ligand to metal due to the complexation (L-MCT) for H_2_Lph and VOLph were located at 396 and 389 nm, respectively, in the visible region of radiation. Specifically, for VOLph and CuLph, in the low-energy area, there were absorption bands at 441 and 448 nm due to the transition of the *d*→*d* orbitals, respectively. This result could indicate that vanadium and copper ions in VOLph and CuLph have 4+ charge with 3*d*^1^ and 2+ charge with 3*d*^9^ electronic distributions, without any regard for the probable oxidation of the V^4+^ ion to the V^5+^ ion in its coordinated compound (VOLph) [36]. The electronic spectroscopy results for the VOLph and CuLph solutions of the *d*→*d* type were attributable to the ^2^T_2g_ → ^2^E_g_ and ^2^B_1g_ (d_z2_) → ^2^E_g_ (d_xz_ and d_yz_) types of electronic transition in the visible region.

#### 2.1.3. FT-IR Spectroscopy

**Figure 3:**
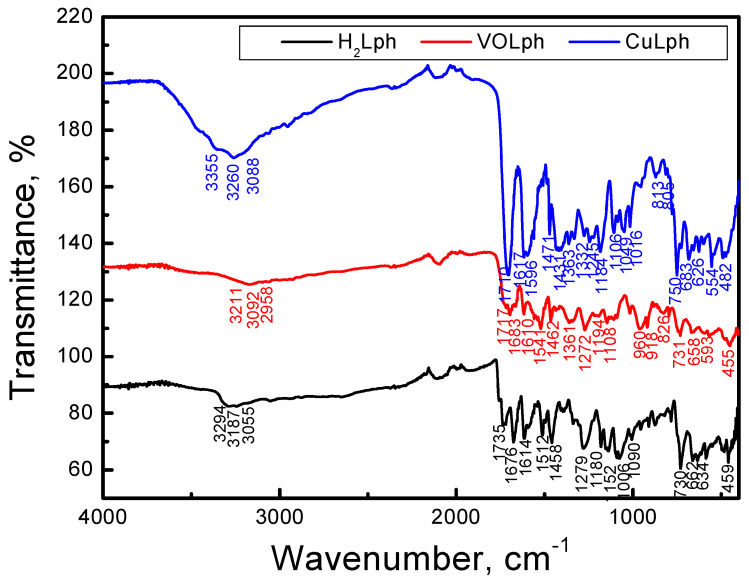
FTIR spectroscopic scans for H_2_Lph, VOLph, and CuLph at room temperature.

The samples of H_2_Lph, VOLph, and CuLph were examined using the FT-IR spectra, as shown in Figure 3. The vibrating spectral absorption (as broad bands) for the two N–H bonds of H_2_Lph, of the diisatin ring and the hydrazone chain, was found to have high frequencies at 3294 and 3187 cm^−1^, respectively. H_2_Lph was found to have a keto structure (referring to its stable form) depending on the high intermolecular resonant frequencies (Scheme 2). The absorption vibrating bands of the two typical bonds of the N–H group (for the isatin rings) in free H_2_Lph shifted slightly after coordinating with VO^2+^ and Cu^2+^ ions, being observed at 3211 and 3260 cm^−1^ for VOLph and CuLph, respectively. Furthermore, the spectral absorption band of the two amido (N–H) bonds of the hydrazone terephethlyl moiety disappeared after the complexation due to their coordination with VO^2+^ and Cu^2+^ ions through the enolic tautomer of H_2_Lph (Scheme 2).

The strong absorption band for the >C=O (carbonyl group) in the isatin ring was observed at 1676 cm^−1^ in H_2_Lph and further appeared for VOLph and CuLph at 1617 and 1610 cm^−1^, respectively. Consequently, a significant hypsochromic shift was observed in the IR band for the stretching and bonding of the >C=O group, exhibiting the bonding of the VO^2+^ and Cu^2+^ ions to the oxygen lone pairs to form VOLph and CuLph chelates, respectively [37]. The wavelength value for the >C=O groups in the keto isomer of H_2_Lph was located at 1735 cm^−1^, which disappeared after the ligand coordination with VO^2+^ and Cu^2+^ ions [42]. The newly formed >C=N– double bond of the enolic isomer of the organo-bonded ligand was observed at 1541 and 1596 cm^−1^ in VOLph and CuLph, respectively [29,36], indicating the coordination behavior of H_2_Lph with VO^2+^ and Cu^2+^ ions within the labile deprotonated anionic –OH group in the enolic tautomer (Scheme 2). Also, the IR spectra for the imino >C=N– groups in H_2_Lph were located at 1512 cm^−1^, as a moderate absorption band, which clearly shifted in VOLph and CuLph to be located at 1462 and 1471 cm^−1^, due to the coordination of H_2_Lph with VO^2+^ and Cu^2+^ ions within the nitrogen atom, respectively [32,37]. The absorption bands for the hydrocarbon chains of C–H bonding of the aliphatic groups for the acetyl acetonate group in the VOLph complex were observed at 2958 cm^−1^ as broad weak bands. The terephathlyl aryl ring displayed absorption spectra for the C–H bonding in H_2_Lph, VOLph, and CuLph at 3055, 3092, and 3088 cm^−1^, respectively. For VOLph and CuLph, new significant vibrational bands were located at 918 (V=O, oxo-ligand), 731 (O→V), 658 (O→V) and 593 cm^−1^ (N→V), and also at 750 (O→Cu), 683 (O→Cu), 554 (N→Cu) and 482 (Cl→Cu) cm^−1^, referring to the absorption stretching of the coordination bonds, respectively.

#### 2.1.4. EI-Mass Spectroscopy

**Figure 4:**
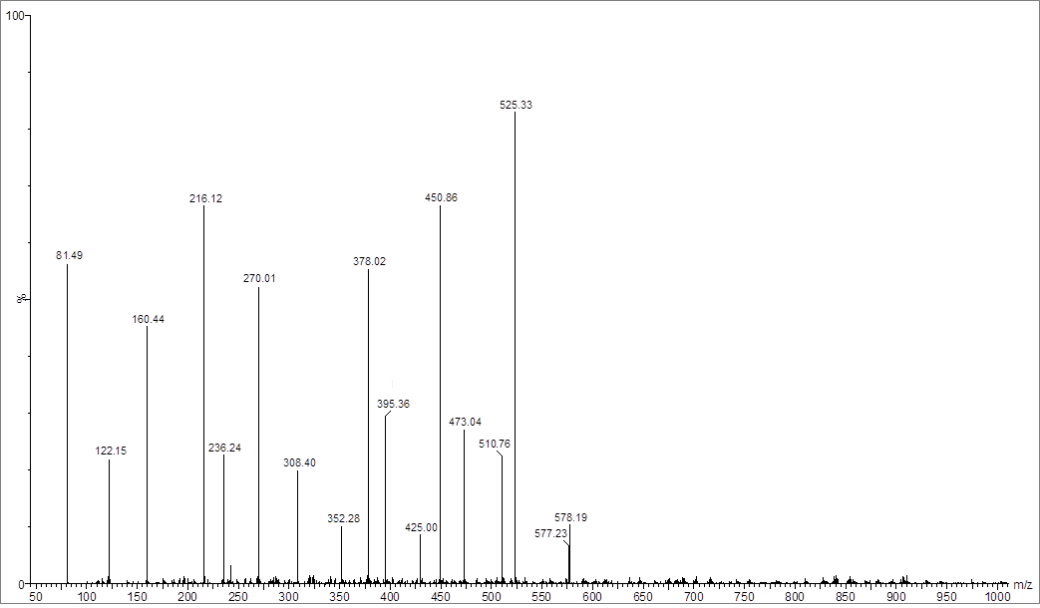
EI-Mass spectra of CuLph at 25 °C in DMF media.

The EI-Mass spectra for the diluted solutions of H_2_Lph, VOLph, and CuLph are shown in Figures S5 and S6 and Figure 4, respectively. Notably, H_2_Lph displayed the most significant base peaks at 453.90 and 452.46 *m*/*z* for the mass fraction for the [HL + 1] mode (C_24_H_9_N_6_O_4_), and also at 348.16 (C_16_H_8_N_6_O_4_), 300.72 (C_12_H_8_N_6_O_4_), 272.85 (C_12_H_8_N_4_O_4_), 216.17 (C_10_H_8_N_4_O_2_), 190.78 (C_9_H_7_N_3_O_2_), 162.01 (C_8_H_6_N_2_O_2_) and 132.90 *m*/*z* (C_8_H_4_O_2_) for the other H_2_Lph mass fractions (Figure S5). VOLph exhibited base peaks at 783.07 and 782.59 for the mass portion for [ML + 1] (C_34_H_30_N_6_O_10_V_2_), i.e., the positive mode, and also at 683.31 (C_29_H_21_N_6_O_8_V_2_), 584.03 (C_24_H_14_N_6_O_6_V_2_), 532.27 (C_20_H_10_N_6_O_6_V_2_), 482.23 (C_16_H_8_N_6_O_6_V_2_), 434.32 (C_12_H_7_N_6_O_6_V_2_), 402.08 (C_12_H_6_N_4_O_6_V_2_), 243.20 (C_9_H_6_N_2_O_3_V), and 164.18 *m*/*z* (C_3_H_2_N_2_O_3_V) for the residual mass portion fragmentation of VOLph (Figure S6). The EI-Mass spectra for CuLph were reported firstly with a remarkable base peak at 578.19 and 577.23 *m*/*z* for [ML − 2Cl^−^] (C_24_H_14_Cu_2_N_6_O_4_), and at 525.33 (C_20_H_10_Cu_2_N_6_O_4_), 473.04 (C_16_H_8_Cu_2_N_6_O_4_), 450.86 (C_14_H_7_Cu_2_N_6_O_4_), 425.00 (C_12_H_6_Cu_2_N_6_O_4_), 395.36 (C_12_H_5_Cu_2_N_4_O_4_), 236.29 (C_9_H_4_CuN_2_O_2_) and 160.44 *m*/*z* (C_3_H_2_CuN_2_O_2_) for the additional mass fractional peaks (Figure 4).

#### 2.1.5. Stability of the Studied Compounds

The biomolecular interactions of a metallo-drug depend on its stability in the studied reaction media, i.e., DMSO [7,8]. The concentration of the ligand and the two complexes was established by dissolving them in DMSO (1.0 × 10^−4^ mol dm^−3^). The stability of H_2_Lph, VOLph, and CuLph was checked by determining the absorbance values at the optimum absorption wavelength in the visible area for the electronic spectra in a time-dependent manner at room temperature. The results exhibit that H_2_Lph, VOLph, and CuLph compounds were stable for a long time, up to 3 days. The absorbance values for H_2_Lph, VOLph, and CuLph (at 396, 441, and 448 nm) were measured at the beginning of the stability tests as 0.37, 0.41, and 0.44, respectively. After 3 days, the absorbance values were obtained as 0.36, 0.41, and 0.42, respectively. However, after 4 days, these values were 0.24, 0.29, and 0.31, respectively. Therefore, the maximum stability of the current compounds in DMSO was observed at 3 days.

### 2.2. Biological Studies

#### 2.2.1. Antimicrobial Assays

Based on the growth inhibition of three fungal and three bacterial types, the bioaction potential of MLph chelates (VOLph and CuLph) was investigated compared to that of H_2_Lph (as the free ligand) in order to explore the pivoting of Pd^2+^ and VO^2+^ ions in their chelates. The three types of bacteria used were *S. marcescens*, *E. coli*, and *S*. *aureus* and the three types of fungi used were *C. albicans*, *A. flavus*, and *T. rubrum*. The data obtained for the progression of the inhibition (based on the area of the inhibition zone) of the growth of the examined microbial cells (in mm) for H_2_Lph, VOLph, and CuLph were explored and are listed in Table 2, and were compared to the results for gentamicin and fluconazole (the most popular antibiotics) [10,43]. Consequently, regarding each of the bacterial strains, H_2_Lph, VOLph, and CuLph established a zone of inhibition of the growth of *S. marcescens* which was 19, 36, and 35 mm in size, a zone of inhibition of the growth of *E. coli* which was 18, 30, and 29 mm in size, and a zone of inhibition of the growth of *S*. *aureus* which was 19, 39 and 38 mm in size, respectively. For the fungal strains, H_2_Lph, VOLph, and CuLph established a zone of inhibition of the growth of *C. albicans* which was 17, 25, and 26 mm in size of the growth of *C. albicans*, a zone of inhibition of the growth of *A. flavus* which was 13, 20, and 20 mm in size, and a zone of inhibition of the growth of *T. rubrum* which was 16, 28, and 27 mm in size (Table 2). The inhibition action of VOLph and CuLph against both bacterial and fungal growth was higher than that of H_2_Lph (the uncoordinated ligand) [6], especially against *E. coli* and *S*. *aureus* [42].

**Figure 5:**
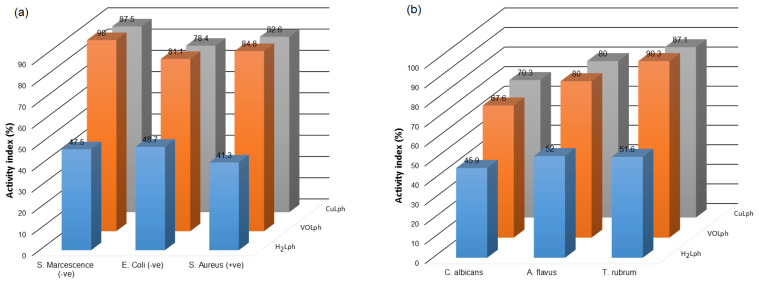
The activity index percentages (*A*%) for (**a**) the bacterial strains and (**b**) the fungal strains after treatment with H_2_Lph, VOLph, and CuLph (with a concentration of 20 μM in DMSO).

Equation (3) was utilized to derive *A*%, the activity index as a percentage (%) for the antimicrobial potential of H_2_Lph, VOLph, and CuLph, listed in Tables S1 and S2 (Figure 5a,b). The *A*% for VOLph and CuLph chelates against the Gram-negative bacterial growth was found to be 90.0 and 87.5% for *S. marcescens* and 81.1 and 78.4% for *E. coli*, respectively, with a notably improved inhibition action over that of H_2_Lhm, (with *A*% = 47.5 and 48.7%, respectively). Similarly, for the Gram-positive bacteria, i.e., *S*. *aureus*, *A*% = 41.3, 84.8, and 82.6% for H_2_Lph, VOLph, and CuLph, respectively (Figure 5a). The results show that the *A*% for H_2_Lph, VOLph, and CuLph against Gram-negative bacteria was high than against Gram-positive bacteria, as observed in Figure 5a,b. The differences in the cell wall structure for Gram-positive and Gram-negative bacteria could be the reason for this [43]. For the assay of fungal inhibition action, an *A*% of 45.9, 67.6 and 70.3% was observed for the inhibition of *C. albicans* growth, an *A*% of 52.0, 80.0 and 80.0% was observed for the inhibition of *A. flavus* growth, and an *A*% of 51.6, 90.3 and 87.1% was observed for the inhibition of *T. rubrum* growth, respectively (Figure 5b). The fungal strains’ results show similar significant effects to those for the bacterial strains after treatment with H_2_Lph, VOLph and CuLph.

Notably, the MIC values displayed the lowest concentration for H_2_Lph, VOLph, and CuLph solutions for the inhibition of the current microbial growth (Table 3). The obtained MIC values were found in the region of 5.80–6.25 μM (H_2_Lph), 1.00–1.50 μM (VOLph), and 1.00–1.75 μM (CuLph) for the bacterial series (*S. marcescens*, *E. coli* and *S*. *aureus*), respectively. The MIC values were in the region of 6.25–7.05 μM (H_2_Lph), 1.25–1.50 μM (VOLph), and 1.25–1.75 μM (CuLph) for the fungal series (*C. albicans*, *A. flavus*, and *T. rubrum*), respectively. Both the *A*% and the MIC values of the antimicrobial studies (Figure 5 and Table 3) for the VOLph and CuLph complexes demonstrated a more significant effect than that for H_2_Lph (as the free ligand) [9]. The hydrophobicity of the designed organometallic drugs demonstrates that they could be preferred candidates for all routes of drug administration depending on the degree of their dissolution and solubility [29]. The improved antimicrobial action of MLph complexes compared to H_2_Lph, as a free ligand, was identified due to the presence of two M^2+^ ions (VO^2+^ and Cu^2+^ ions) [42,43]. Modified bioreactivity for the inhibition action was illustrated for both VOLph and CuLph, superior to that of H_2_Lph, establishing the effect of the two central M^4+^ ions presented in [44]. For VOLph and CuLph complexes, the penetration action into the microbe cell walls was accomplished through a significant distribution process, which could effectively react with the blocking of the synthesized growth protein to a greater extent than the same process with H_2_Lph [29]. This protein was responsible for functionalizing the respiratory system of the microbial cell, and consequently, the respiration metabolism could be blocked and then the cell growth was completely stopped due to the lipophilicity of the synthetic compounds [45]. Accordingly, VOLph and CuLph complexes with more lipophilic and hydrophobic effects could reduce lipid metabolism to a greater extent than the free H_2_Lhm ligand [24]. These improved features of VOLph and CuLph complexes could be interpreted by considering the chelating theory of Tweedy’s effect (i.e., the neutralization) of V^4+^ and Cu^2+^ cations (i.e., the positive species) with H_2_Lph (the negatively charged species) in their complexes. In other words, V^4+^ and Cu^2+^ ions were neutralized with the negatively charged coordinated ligand (H_2_Lph) through the overlapping of the empty orbitals of nV^4+^ and Cu^2+^ ions with the filled orbitals with negative charges and the electron pairs of the heteroatoms (*N*,*O*-atoms) [46]. The neutralized species of VOLph and CuLph complexes diminished their polar nature compared to H_2_Lph, and hence improved their lipophilicity and hydrophobicity. The enhanced penetrating potential of VOLph compared to that of CuLph was due to the Lewis acidic feature, lipophilicity (hydrophobicity), and electronegativity of the V^4+^ ion with high valence (attached to an oxo-ligand) compared to that of the cationic Cu^2+^ ion (with a low valence) in their corresponding complexes, respectively [29,42].

The type of M^n+^ ion and its charge in the organo-coordinated complexes displayed a major effect on the ribosomes and impermeability of the microbial cells [47]. Both VOLph and CuLph chelates displayed higher potential as antimicrobial reagents compared to the free H_2_Lph ligand [10,29]. However, the functionalized organic groups, especially the hydrazone chain in H_2_Lph and its VOLph and CuLph chelates, could form hydrogen bonding with the cellular constituents within the active centers. This bonding could cause significant interference with normal cellular processes [48].

Fluconazole and ciprofloxacin (well-known antibiotic drugs) were employed in a comparative study with H_2_Lph, VOLph, and CuLph. H_2_Lph, VOLph, and CuLph demonstrated less effectiveness compared to these antibiotic drugs (fluconazole and ciprofloxacin), as listed in Table 2. Furthermore, DMSO was used in the same study as a reference control solvent, and did not exhibit any considerable inhibiting action against the titled microorganisms’ growth [8].

In order to understand the role of the M^n+^ metal ion in its organometallic chelate in inhibiting microbial growth, the inhibiting potential of some analogs of mono- and bimetallic homoleptic hydrazone chelates from our previous work (in mm) against the studied microbes is presented in Table 4, in order to perform a comparison with the listed examples of mononuclear V^4+^O- and Cu^2+^-chelates [49,50]. The current bimetallic VOLph and CuLph chelates have a higher inhibition power than the monometallic analogs (Table 4), based on their modified lipophilicity and electronegativity [29,49,50]. Also, the reactivity of a bimetallic zinc (II) analog (succinate derivative) was less than that for the VOLph and CuLph complexes due to the low redox action (redox inert) of the Zn^2+^ ion in the reported analog [28].

The biochemical features of VOLph and CuLph exhibited some improvement in microbial inhibition action compared to a succinate derivative [29], and this could be attributed to their more lipophilic character due to the presence of the phenyl ring as a π-interacting system.

#### 2.2.2. Antiproliferative Action

**Figure 6:**
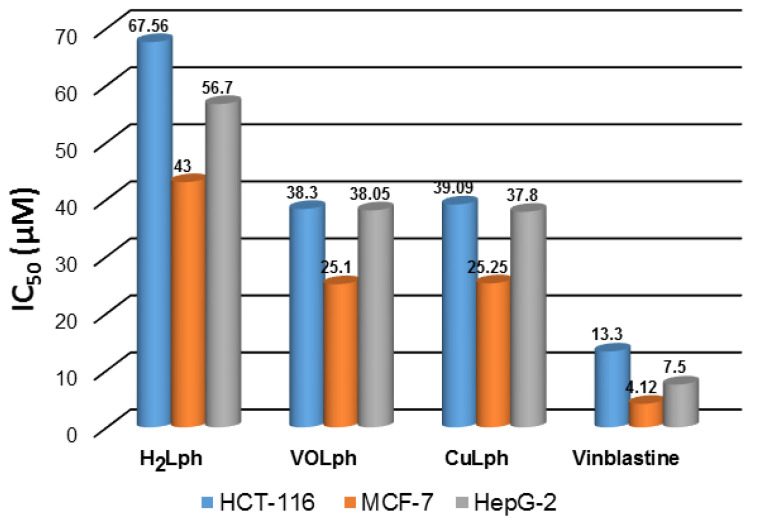
The antiproliferative index (IC_50_) for H_2_Lph, VOLph, and CuLph reagents versus the current human cancer cell lines.

The antitumoral activity of H_2_Lph, VOLph, and CuLph was examined based on their ability to inhibit the growth of some common human cancer cell lines, namely three tumors entitled HepG2, MCF-7, and HCT-116, using an informative tool, SRB (sulforhodamine-B-stain). The most popular antitumor drug (vinblastine) was used to compare its activity with that of H_2_Lph, VOLph, and CuLph. Notably, the antitumor activity of H_2_Lph, VOLph, and CuLph was exhibited by the IC_50_ (in µM) for inhibiting the growth of human cancer or normal cell lines, as given in Table S3 and represented in Figure 6, in which IC_50_ concentrations were obtained from Equation (2).

Observably, the IC_50_ of H_2_Lph was obtained as 67.56, 56.75, and 43.00 μM against HCT-116, MCF-7, and HepG-2 growth, respectively. On the other hand, the IC_50_ diminished obviously with VOLph and CuLph to 38.30 and 39.09 μM against the growth of HCT-116, to 25.10 and 25.25 μM against the growth of MCF-7, and finally to 38.05 and 37.08 μM against the growth of HepG-2, respectively (Figure 6). More significant antitumoral effects could be observed for the free H_2_Lph ligand, agreeing with the antimicrobial results. Hence, the antitumor study demonstrated the influence of the central metal ion on Tweedy’s chelation effect to the bioaction of H_2_Lph in its coordinated form with VO^2+^ and Cu^2+^ ions in VOLph and CuLph, respectively (taking into account their improved Lewis acidity and lipophilic and hydrophobic features) [51].

The modified lipophilic feature could improve the antiproliferative potential of VOLph and CuLph chelates compared to H_2_Lph (i.e., free ligand), referring to the role of the chelation effect, i.e., Tweedy’s theory of chelation, as represented for the bacterial and fungal studies [29,51,52]. The higher activity of VOLph than CuLph was interpreted as being due to the more highly lipophilic character of VOLph, referring to the more positively charged O=V^4+^ ion with high electrophilicity compared to that of CuLph with a less positively charged Cu^2+^ ion with lower electrophilicity, as demonstrated by the IC_50_ values (neutralization effect) [8,46,47]. However, H_2_Lph, VOLph, and CuLph have less antiproliferative action compared to the anticancer drug vinblastine.

The selectivity index refers to the tumor specificity of the studied compounds versus the human cancer cell lines and the normal cells (WI-38) [53], which is presented in Table 5. For the selectivity index, both complexes exhibited a higher selectivity index than their free ligand (H_2_Lph), based on the presented central metal ion. VOLph and CuLph displayed less toxic effects on WI-38 normal cells, which is important for developing new candidates as anticancer drugs. The obtained selectivity results were akin to those of other reported vanadyl and copper chelates [29,51,54,55]. Additionally, the selectivity index was highly appreciable for VOLph, greater than that of CuLph, which was due to the effect of the more electronegative VO^2+^ ion compared to that of the Cu^2+^ ion in VOLph and CuLph, respectively. So, VOLph was suggested to be more selective toward cancer cells than CuLph, and hence it could be safer versus the normal cell populations.

#### 2.2.3. ctDNA Action

##### Electronic Spectroscopic Studies

**Figure 7:**
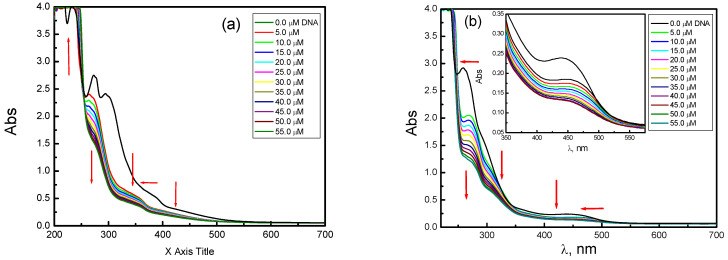
The spectral changes for the electronic transitions for (**a**) VOLph and (**b**) CuLph solutions with [DNA] of different *ct*DNA concentrations in DMSO, in μM (at 25 °C).

For the analysis of the biochemical interaction behavior of H_2_Lph, VOLph, and CuLph with a *ct*DNA solution (calf thymus DNA), defined prepared DMSO solutions of H_2_Lph, VOLph, and CuLph were examined with *ct*DNA solutions spectrophotometrically at a controlled pH [33,34]. The detected absorption (A) at λ_max_ of the characteristic electronic absorption bands in both the ultraviolet and visible regions for the DMSO solutions of H_2_Lph, VOLph, or CuLph with different concentrations of *ct*DNA (in DMSO) was measured and is represented in Figure 7a,b for VOLph and CuLph and Figure S7 for H_2_Lph. The significant shifts and decays (∆*n*) were recorded to distinguish the type and nature of chromism, *K*_b_ (the intrinsic binding constant), and $\Delta G_{b}^{\neq }$ (the Gibbs free energy) for the interacting mode of H_2_Lph, VOLph, and CuLph with *ct*DNA, based on Equations (5) and (6), as shown in Figure 7a,b and Figure S7 (Table 6) [29].

Consequently, the recorded shifts and decays for the absorption bands in the UV region, as shown in Table 6, for H_2_Lph, VOLph, and CuLph solutions in DMSO with increasing *ct*DNA concentrations were employed to establish the change in the electronic absorption bands of the π/n→π* modes. H_2_Lph, VOLph, and CuLph demonstrated obvious shifts and decays of the π→π* transition band from 251, 296, and 262 nm to 263, 270, and 275 nm, with ∆*n* = 22, 26, and 14, respectively. Similarly, the n→π* transition displayed an obvious shift for the free ligand H_2_Lph from 340 nm to 333 nm (with ∆*n* = 7), which was not observed for the corresponding VOLph and CuLph complexes. Such shifts and decays for the π→π* transition, as well as the n→π* transition (in the uncolored area), for H_2_Lph, VOLph, and CuLph were obtained based on the significant interacting ability of the π-aryl and dihydrazine chromophore of the organic substituents through the covalence mode of interaction [10]. Furthermore, the LCT and M-LCT transitions bands were observed to shift and decay from 396, 389, and 300 nm to 380, 347, and 311 nm with ∆*n* = 41, 42, and 11 for H_2_Lph, VOLph, and CuLph, respectively, in the visible region (Table 6). Additionally, the *d*-*d* transition bands for VOLph and CuLph shifted from 441 and 448 nm to 428 and 453 nm, with ∆*n* = 13 and 5, respectively. All the color changes of the spectral bands for H_2_Lph, VOLph, and CuLph (i.e., shift in bathochromic type) were due to the non-covalence of the interaction with *ct*DNA [55,56].

**Figure 8:**
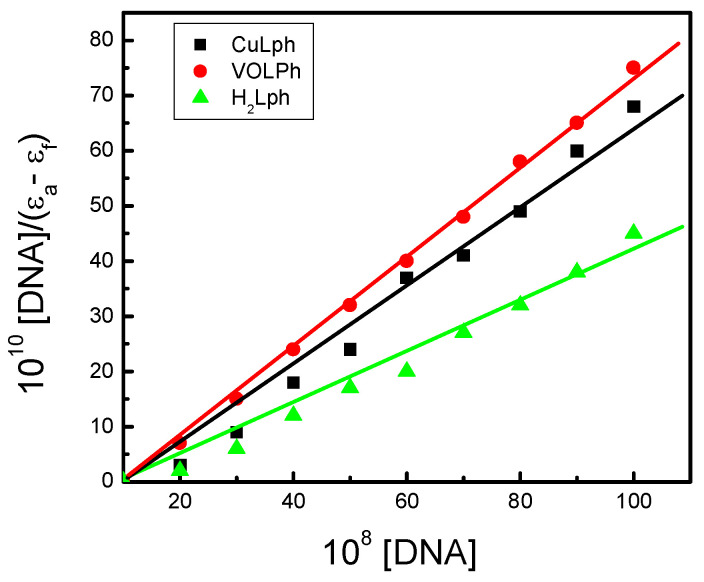
The plot of [DNA]/(*ε*_a_ − *ε*_b_) against the [DNA] of mixed solutions of *ct*DNA with H_2_Lph, CuLph and VOLph at 25 °C.

**Scheme 3:**
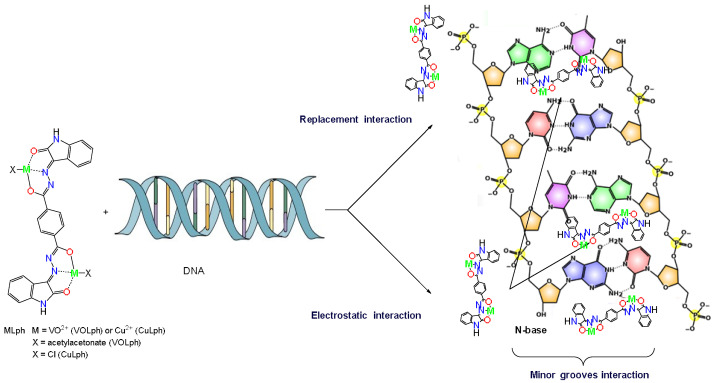
The mode and type of interaction of VOLph and CuLph chelate with *ct*DNA.

The *K*_b_ and $\Delta G_{b}^{\neq }$ values were significantly important for the assessment of the interacting strength and bonding between H_2_Lph, VOLph auLph, and *ct*DNA (as derived from Figure 8). They were found to be 12.41, 16.31, and 16.04 × 10^7^ mol^−1^ dm^3^ (for *K*_b_) and −44.05, −47.11 and −46.89 kJ mol^−1^ (for $\Delta G_{b}^{\neq }$), respectively, as listed in Table 6. Additionally, the mode of chromism for the current study was determined, depending on Equation (5), in order to establish the type of binding for the interacting H_2_Lph, VOLph, and CuLph with *ct*DNA. In particular, the observed absorption spectral bands for the molecules of H_2_Lph, VOLph, or CuLph with *ct*DNA solutions were attributed to their hypochromic features (no detectable hyperchromic character). Hence, the hypochromic character was illustrated for a significant interaction type of *ct*DNA with H_2_Lph, VOLph, or CuLph. On the other hand, the hyperchromic mode, could generally exhibit a distorted mode for the double helix *ct*DNA interaction with specific molecular species. An observable interaction for H_2_Lph, VOLph, and CuLph with the *ct*DNA helix was reported here according to their hypochromic interaction effects. There was no observable damage to the *ct*DNA double helix when reacted with H_2_Lph, VOLph, or CuLph species, as shown in Scheme 3 [57].

The interaction mode of such types of compounds, such as organic and metallo-organic complexes with *ct*DNA could be either non-covalent and/or covalent. The non-covalent modes are reversible without destructive effects on the DNA, whereas the covalence interactions are irreversible and exhibit observable destructive effects against DNA depending on the degree of interaction [58]. The three major types of non-covalent interactions are represented here as electrostatic interactions, groove binding, and intercalation binding modes. These could be due to the interaction of the positive charge of metal ions (VO^2+^ and Cu^2+^ in VOLph and CuLph, respectively) with the negative charge of the phosphate scaffold of the interacting *ct*DNA through electrostatic interactions [59]. The covalent mode was accomplished by ionic, hydrogen, and van der Waals interactions [29]. Such interactions caused remarkable shifts and decays in the colored bands of LCT, MLCT, and *d*-*d* transition in the spectroscopic studies, as shown in Figure 7 for the current compounds. When the H_2_Lph, VOLph, or CuLph molecules interacted with the nitrogenous base pairs of the *ct*DNA molecule, they could interact through forces of the covalent mode, i.e., ionic, hydrogen, and van Der Waals bonding types [60]. Thus, the interaction of H_2_Lph, VOLph, or CuLph with *ct*DNA within the covalent mode could cause distinguished shifts and decays for the spectral bands in the UV area, i.e., π→π* and n→π* transitions. In this context, it was clear that there was no distortion of the *ct*DNA molecule due to its interaction with the H_2_Lph, VOLph, or CuLph molecule, so there was no probability of an intercalative binding mode. When intercalative binding takes place, it can distort the conformation of DNA [60]. On the other hand, when the H_2_Lph, VOLph, or CuLph molecule fitted within the electrostatic or minor grooves mode through hydrogen bonding or van der Waals interactions, no significant distorting effects on the *ct*DNA chain were detected [61]. Moreover, the most attractive mode of interaction was the replacement mode, which was significantly observed for such types of metallorganic complexes, such as VOLph and CuLph [10]. The labile coordinated organic solvent molecule or less stable coordinated species could be replaced by the *ct*DNA molecule to bind to the central metal ion (VO^2+^ or Cu^2+^ ions in VOLph or CuLph, respectively), as shown in Scheme 3 [29,49,50]. Such results agree with the hypochromic features of the current compounds.

The interaction of VOLph and CuLph with the *ct*DNA solution was higher than that of their free H_2_Lph ligand with an order of VOLph > CuLph, as a result of the *K*_b_ and $\Delta G_{b}^{\neq }$ values in Table 6. This could be interpreted based on the type and nature of the coordinated metal ion (VO^2+^ and Cu^2+^ ions). Hence, VO^2+^ and Cu^2+^ ions with an enhanced electrophilic feature in VOLph and CuLph, respectively, could enable their molecules to bind via an additional replacement mode in parallel with the traditional modes (the groove binding and electrostatic interactions) compared to that of the free ligand H_2_Lph [56]. Interestingly, the type of M^2+^ ion in both the VOLph and CuLph chelates causes a distinguished difference in their effectiveness. The more lipophilic character of VOLph compared to that of CuLph, due to the more electrophilic feature of the VO^2+^ ion with a high oxidation state and an oxo-ligand, could enhance its action with *ct*DNA compared to that of the Cu^2+^ ion in CuLph [57].

##### Viscosity Assay

**Figure 9:**
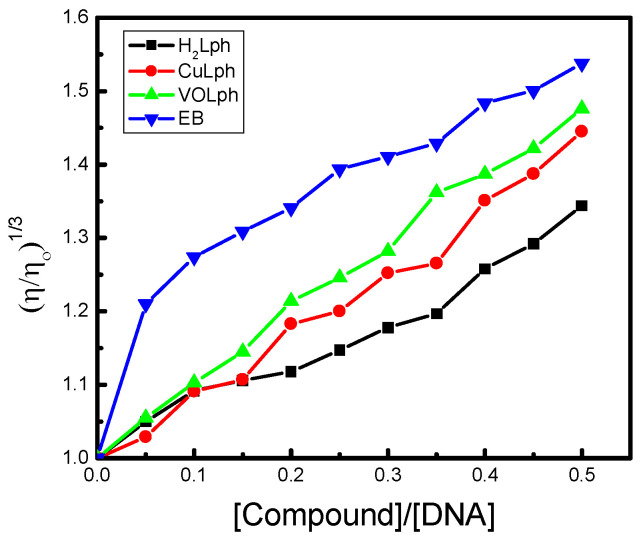
At room temperature, the effect of H_2_Lph, VOLph, and CuLph solutions (with various concentrations) on the viscosity of a *ct*DNA solution in DMSO.

As a factor of biological interest related to H_2_Lph, VOLph, and CuLph, a study to establish the interacting mode with *ct*DNA was performed. A change in the *ct*DNA solution’s viscosity was observed when it was mixed with H_2_Lph, VOLph or CuLph solutions, based on the derived data from Equations (6) and (7). The observed viscosity increase for the *ct*DNA solution was established from the plotted graph of the molar ratios of [Compound]/[DNA] for H_2_Lph, VOLph, and CuLph concentrations to those of the *ct*DNA concentrations against the (η/η_o_)^1/3^ values, as displayed in Figure 9 [56]. The enhancement of *ct*DNA viscosity with the referenced EB, ethidium bromide, was also displayed in order to compare its reactivity with those of H_2_Lph, VOLph, and CuLph.

The observed increase in the viscosity of *ct*DNA with the H_2_Lph, VOLph, or CuLph solutions was lower than that observed for the value achieve when mixing the reagents, i.e., EB, with *ct*DNA, demonstrating EB’s more significant interacting behavior with *ct*DNA (Figure 9). Consequently, the progressing action of the separated *N*-base pair of the *ct*DNA double helix could clearly explain the prolonged double helix of *ct*DNA, causing a remarkable enhancement of the *ct*NDA viscosity with a distinguished interacting action with H_2_Lph, VOLph or CuLph [43]. Accordingly, both the VOLph and the CuLph complex exhibited further modified interacting features in relation to the *ct*DNA solution compared to the behavior of H_2_Lph (the free ligand). These results are supported by the absorption spectral studies following the covalent/non-covalent types of interacting modes. Additionally, regarding the non-covalent mode of interaction, VO^2+,^ and Cu^2+^ ions displayed a significant role in modifying and developing the bioreactivity of their corresponding MLph chelates’ interaction with *ct*DNA molecules (Scheme 3). The lipophilicity of VOLph or CuLph compared to that of their free H_2_Lph ligand could notably enhance their interacting reactivity with *ct*DNA molecules [29]. In addition, the electronegativity and Lewis acidity behavior of VO^2+^ and Cu^2+^ ions in their complexes could promote the lipophilic character of VOLph or CuLph and enhance their interaction with *ct*DNA compared to H_2_Lph, as described in Scheme 3 [62].

##### Gel Electrophoresis

**Figure 10:**
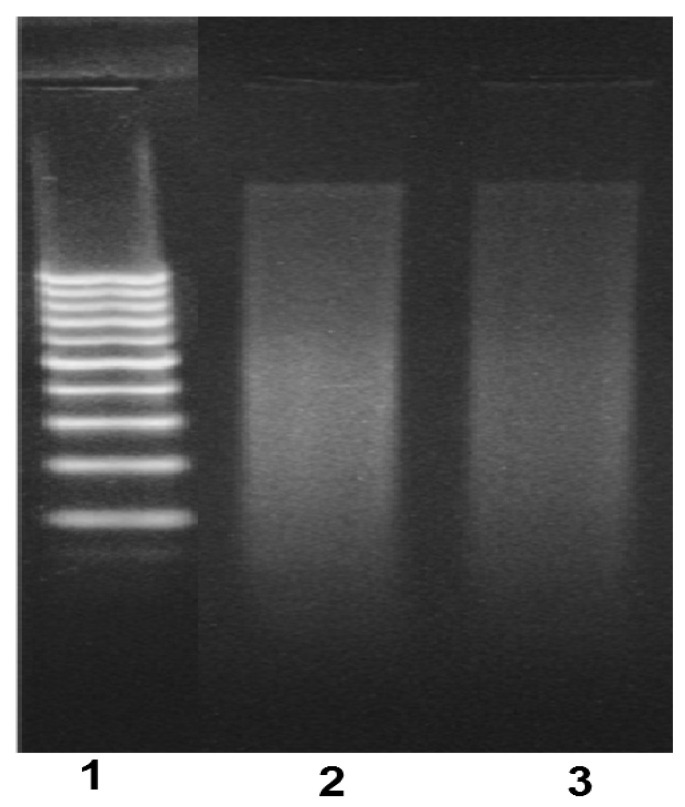
Gel electrophoresis of (Line 1) *ct*DNA, (Line 2) *ct*DNA + VOLph, and (Line 3) *ct*DNA + CuLph.

Gel electrophoresis is a valuable tool to observe the main change in the *ct*DNA’s characteristics due to its interaction with VOLph and CuLph [55,56]. The obtained gel phase after electrophoresis demonstrated that the *ct*DNA intensity color after the treatment with VOLph or CuLph chelate partially changed and decayed, as shown in Figure 10. Based on *ct*DNA’s binding reactivity with the current VOLph and CuLph complexes, it can be concluded that the studied complexes inhibit the growth of a pathogenic organism by interacting with the genome of *ct*DNA [54,63].

## 3. Experimental

### 3.1. Materials and Methods

A description of the devices that were used in the current work is represented in the Supplementary Materials.

### 3.2. Synthesis

#### 3.2.1. Synthesis of H_2_Lph Organo-Ligand

As previously reported for aroyl-dihydrazide synthesis [28,29], isatin was condensed directly to terephathlyl dihydrazide by mixing 1.47 g of 10 mmol isatin in 50 mL MeOH in a round flask containing 0.97 g of 5 mmol terephthalic dihydrazide in 30 mL of MeOH. The obtained mixed solution of the reactants was heated at 80 °C with magnetic stirring for 4 h. The product was monitored by the TLC, with the formation of an orange precipitate of H_2_Lph, which was then filtered using filter paper. The collected precipitate was purified by means of recrystallization in ethanol to produce 1.59 g of pure orange powder of H_2_Lph, with a 70% yield, as reported [30].

^1^HNMR spectra of H_2_Lph: δ = 6.91 (d, ^3^*J* = 6.8 Hz, 2H), 6.96 (d, ^3^*J* = 6.9 Hz, 2H), 7.05 (t, ^3^*J* = 7.0 Hz, 2H), 7.10 (d, ^3^*J* = 6.9 Hz, 2H), 7.38 (d, ^3^*J* = 7.2 and 7.0 Hz, 4H), 7.57 (d, ^3^*J* = 6.8 Hz, 2H), 8.12 (d, ^3^*J* = 7.0 Hz, 2H), 10.70 (s, 2H, NH) and 11.28 ppm (s, 2H, NH). For the dienolic form: δ = 6.92 (d, ^3^*J* = 7.0 Hz), 7.10 (d, ^3^*J* = 7.0 Hz) and 12.55 ppm (s, NH) (Figure S1).

^13^CNMR spectra of H_2_Lph: δ = 111.00 (CH), 111.58 (C_q_), 115.81 (CH), 120.35 (C_q_), 122.11 (CH), 123.03 (CH), 126.56 (CH), 131.89 (C_q_), 132.85 (C_q_), 150.02 (C_q_, C=N), 162.81 (C_q_, C=O) and 165.11 ppm (C_q_, C=O) (Figure S2).

#### 3.2.2. Preparation of MLph Chelates

The free ligand H_2_Lph (0.90 g, 2.0 mmol) in methanol (40 mL) was dropped gently into a round flask containing 1.06 g or 0.68 g of oxovanadium acetylacetonate (VO(acac)_2_) or copper chloride dihydrate (CuOCl_2_·2H_2_O) (4.0 mmol) in water (40.0 mL) at room temperature, respectively. The reaction of complexation was accomplished with stirring (magnetically) and heating (at 80 °C) for approximately 3.5 h and monitored by the TLC. After finishing the complexation reaction, solvents were removed under vacuum. The remaining impure solid of the target MLph complex (VOLph and CuLph) was purified by means of crystallization using methanol followed by washing the pure complex with *n*-hexane to produce 1.05 and 0.92 g of VOLph and CuLph with a 68% and 71% yield, respectively. The m.p. was >300 °C for both MLphs.

### 3.3. Biological Studies

#### 3.3.1. Antimicrobial Studies

Using the resazurin method [31,32], as the most viable spectroscopic method, the inhibiting reactivity of H_2_Lph, as a free ligand, and its chelating VOLph or CuLph complex was assessed against the cell growth of three bacterial and three fungal series. The three bacterial series were *E. coli* (*Escherichia coli*) and *S. marcescens* (*Serratia marcescens*), as Gram-negative types, and *S. aureus* (*Staphylococcus aureus*), as a Gram-positive type. The fungal series were *A. flavus* (*Aspergillus flavus*), *C. albicans* (*Candida albicans*), and *T. rubrum* (*Trichophyton rubrum*). The six series of bacteria and fungi were ordered from the American Type Culture Collection (ATCC, Manassas, VA, USA). A solution of the dissolved resazurin tablet with a weight of ~3.70 g in 40 mL of water was prepared. Similarly, a stock fresh solution, as the mother concentrated solution, of H_2_Lph, VOLph, and CuLph was prepared with 20 µM concentration in fresh DMSO. Using the mother solution of H_2_Lph (20 µM), VOLph, and CuLph, 100 µL was poured into the solution of the tablet of resazurin as a dissolved solution in the 96-well plates three times while preparing another empty well (as a referenced reagent). We used 50 µL of sterile nutrient broth as a solution to fill the residual wells. As the standard referenced solution, 10.0 µL of the prepared resazurin solution was mixed under magnetic stirring into the above-prepared wells containing H_2_Lph, VOLph, and CuLph samples. Following that, 10.0 µL of a suspension solution of one of the studied microbes (one of the three bacteria or the three fungi) was supplied to the wells of the obtained resazurin solution with the studied compound (H_2_Lph, VOLph, or CuLph). In the following step, the resulting solution of H_2_Lph, VOLph, and CuLph was mixed with 30 µL of a solution of sterile nutrient broth, as prepared previously [33]. To avoid dehydrated action for the current microorganism series (i.e., bacteria and fungi), all of the examined plates were covered with cling film. For a comparative study, the antibiotic drugs fluconazole and ciprofloxacin were used as the standard positive drugs in the current studies.

#### 3.3.2. Activity Index (A) and Minimal Inhibited Concentration (MIC)

The evaluation of the inhibiting power of H_2_Lph, VOLph, and CuLph versus the growth of the studied microorganisms was performed by establishing the minimal concentration required for inhibition (as the minimal inhibition concentration = MIC). The MIC studies were performed at minimal concentrations of the H_2_Lph, VOLph, and CuLph solutions in fresh DMSO with the highest inhibiting power against the microorganisms’ growth [33]. After finishing the incubation, the incubated microorganisms were disseminated on nutrient agar plates under atmospheric conditions for 3 days at 37 °C. For a period of almost 3 days at 37 °C, the incubation process was performed for H_2_Lph, VOLph, or CuLph with the currently examined microorganisms. Then, the MIC was determined, and the corresponding activity index (A, in percentages) was derived using Equation (1) for H_2_Lph, VOLph, and CuLph:

(1) $$A=\frac{\mathrm{Inhibition}\;\mathrm{zone}\;\left(\mathrm{mm}\right)}{\mathrm{Inhibition}\;\mathrm{zone}\;\mathrm{of}\;\mathrm{standard}\;\mathrm{drug}\;\left(\mathrm{mm}\right)}\times 100$$

#### 3.3.3. Antitumor Assays

Using HepG-2 (hepatocellular carcinoma), HCT-116 (colon carcinoma), and MCF7 (breast adenocarcinoma), as the most famous human cancer cell lines, the antitumor reactivity of H_2_Lph, VOLph, and CuLph was studied by applying the well-known spectrometric methodology [31]. SRB (i.e., sulforhodamine-B-stain) was applied in the spectrometric studies at a specific wavelength (564 nm, the optimized absorption wavelength). The recording of the corresponding absorbance (*A*) was accomplished using a UV/Vis. spectrophotometer, a UV-1800 mode Shimadzu, with a mono-cell holder. With plates of a 96-multiwell model, the culturing of the current human cancer cell lines with 10^4^ cells per well was accomplished, followed by the gentle addition of DMSO solutions of H_2_Lph, VOLph, or CuLph to the cultured wells of the cancer cell lines. The resulting mixed solutions of H_2_Lph, VOLph, or CuLph with the studied human cancer cell line in SRB were incubated in an isolated atmosphere for 48 h at 37 °C. The isolated atmosphere was accomplished by means of CO_2_ gas bubbling with *v*/*v* 15%. The same procedure was followed with vinblastine, which was used as a reference antitumor agent to compare its reactivity with that of the studied H_2_Lph, VOLph, and CuLph compounds. For WI-38, as the normal human fetal lung fibroblast, the previous procedure used for the cancer cell lines was applied for H_2_Lph, VOLph, and CuLph in order to evaluate the selectivity index of the these antitumor reagents. Equation (2) was applied to derive the inhibition potential of H_2_Lph, VOLph, or CuLph against the growth of human cancer and normal cell lines through their effective concentration in μM (IC_50_, the effective concentration of H_2_Lph, VOLph, or CuLph) [34]:

(2) $${\mathrm{IC}}_{50}\left(\mu M\right)=\frac{Control_{OD}-Compound_{OD}}{Control_{OD}}\times 100$$

Free DMSO was used as the standard applied solvent, i.e., the negative reference for determining the valuable inhibition potential of the H_2_Lph, VOLph, or CuLph compounds for the growth of human normal/cancer cell lines.

#### 3.3.4. Nature of Interaction with ctDNA

Various diluted concentrations of *ct*DNA in DMSO at a pH under 7.5 (with NaCl of 50 mM and tris-HCl of 5.0 mM in water) were mixed with defined concentrations of H_2_Lph, VOLph, and CuLph to examine their reactivity at room temperature. Using the mother stock solution of *ct*DNA, the work period for the diluted concentrations was a maximum of 4 d at 4 °C. To examine the presence of proteins in the *ct*DNA source, as a measurement of its purity, the molar absorptivity was estimated at λ_max_ = 260 nm for the sample solution of *ct*DNA, and was found to to be 6600 mol^−1^ cm^−1^, indicating the absence of any contaminated proteins in *ct*DNA. For more accuracy, another test was accomplished for the same purpose, by measuring the absorbance at 260 and 280 nm for the *ct*DNA solution and then calculating the A_260_/A_280_ ratio, which was obtained as 1.88, being between 1.8 and 1.9. This means that the *ct*DNA solution did not contain any contaminated proteins in its pure form. Ethidium bromide solution (EB) in fresh DMSO was also involved in the study at its characterized λ_max_ = 480 nm and ε = 5860 mol^−1^ cm^−1^, as a commonly referenced reagent against *ct*DNA. The bioreactivity of H_2_Lph, VOLph, and CuLph was compared to the reported results of EB. Several concentrations of diluted solutions for H_2_Lph, VOLph, or CuLph in fresh DMSO at room temperature were successfully within less than 0.1% *v*/*v* error. Then, these diluted concentrations of H_2_Lph, VOLph, or CuLph were mixed with 50 mM *ct*DNA solution (at pH 7.5, controlled by 5.0 mM of tris-HCl_(aq)_) and 50 mM NaCl_(aq)_, and then examined using a UV-Vis. Spectrophotometer, the UV-1800 model from Shimadzu.

##### Electronic Spectroscopic Assay

For the diluted concentrations of the *ct*DNA solution (μM) in DMSO (at 25 °C), different mixtures were prepared with a H_2_Lph, VOLph, or CuLph solution (concentration = 5.0 μM in DMSO). The ultraviolet-visible spectra were measured for these mixtures using the Shimadzu UV-1800 spectrophotometric instrument, and then the observed changes (decay-shift) for the characteristic absorption bands for the solutions of H_2_Lph, VOLph, or CuLph were recorded [31]. From the observed changes (decay–shift) for the characteristic spectra for the mixed solutions of *ct*DNA and the solution H_2_Lph, VOLph, or CuLph (at λ_max_), *K*_b_, the binding constant from Equation (3), and $\Delta G_{b}^{\neq }$, standard Gibb’s free energy from Equation (4), were derived and calculated, which refer to the binding nature strength between the *ct*DNA molecule and the interacting H_2_Lph, VOLph or CuLph molecule in the mixed solution. Based on the plotted figures for the [DNA]/(*ε*_a_ − *ε*_b_) versus [*ct*DNA] ratio:

(3) $$\frac{\left[DNA\right]}{\varepsilon_{a}-\varepsilon_{f}}=\frac{\left[DNA\right]}{\varepsilon_{b}-\varepsilon_{f}}+\frac{1}{\left[K_{b}(\varepsilon_{b}-\varepsilon_{f})\right]}$$

(4) $$\Delta G_{b}^{\neq }=-RTlnK_{b}$$

In Equation (3), [*DNA*] represents the different *ct*DNA concentrations in DMSO, *ε_a_*, *ε_f_*, and *ε_b_* are the extinction coefficients for various *ct*DNA solutions with a H_2_Lph, VOLph or CuLph solution, for the various free *ct*DNA solutions (in the absence of the addition of a H_2_Lph, VOLph or CuLph solution), and at the end of the interaction between the *ct*DNA solutions and the H_2_Lph, VOLph or CuLph solution, respectively [32]. *R* and T are defined as the gas constant and absolute temperature, respectively. The nature and type of chromism (in %) of the interacting H_2_Lph, VOLph, or CuLph solution with *ct*DNA solutions in DMSO was deduced using Equation (5):

(5) $$\mathrm{Chromism}\;\left(\%\right)=\frac{A_{free}-A_{bonding}}{A_{free}}$$

where *A_free_* and *A_bonding_* are the absorbance for the H_2_Lph, VOLph, or CuLph solution at λ_max_ without *ct*DNA solutions and with different concentrations of *ct*DNA solutions in DMSO, respectively.

##### Viscosimetric Study

The improved viscosity of the *ct*DNA solution in DMSO (5.0 μM) with different added portions of H_2_Lph, VOLph, or CuLph solutions of various concentrations (0.0–50.0 μM) was measured using an Oswald micro-viscometer. Such processes were achieved under an inert atmosphere, i.e., bubbling N_2_ gas in the *ct*DNA solution with H_2_Lph, VOLph, or CuLph solutions. The enhanced viscosity of the *ct*DNA solution with H_2_Lph, VOLph, or CuLph solutions was deduced using Equation (6) [29]. The *ct*DNA solution’s viscosity without any additives and the *ct*DNA solution’s viscosity with solutions in DMSO for H_2_Lph, VOLph, or CuLph are expressed by *η*^o^ and *η*, respectively.

(6) $$\eta =\frac{t-t^{o}}{t^{o}}$$

With Equation (6), *t* and *t*^o^ represent the fluid time consumed by the mixed solution of *ct*DNA and the H_2_Lph, VOLph, or CuLph solution and the free *ct*DNA solution in DMSO, respectively. Moreover, the figure for the ratio values of (*η*/*η*°)^1/3^ was plotted against the values of *R*, where *R* refers to the interaction degree for *ct*DNA with H_2_Lph, VOLph, or CuLph. *R* was calculated using Equation (7), from the ratio of [comp.]/[*ct*DNA]:

(7) $$R=\frac{\left[ctDNA\right]}{\left[comp.\right]}$$

From Equation (7), [*ct*DNA] and [*comp.*] represent the *ct*DNA solution and the different H_2_Lph, VOLph, or CuLph diluted solutions in DMSO, respectively.

##### Gel Electrophoresis Assay

The binding ability of VOLph and CuLph with *ct*DNA was established using gel electrophoresis [33]. The current chelates were poured into *ct*DNA in an equivalent volume in DMSO with incubation for 1 h (at 37 °C). After that, the *ct*DNA loading dye solution was mixed with the obtained solutions of VOLph or CuLph at a 1:1 ratio of molarities and then poured into the buffer solution of the gel (1%) in TAE. For 60 min, 100 V (as a fixed voltage) was supplied to the resulting mixed solutions. In the end, the gel was imaged under an ultraviolet light source using a transilluminator. The images of the DNA gel were documented using Panasonic DMC-LZ5 Lumix (in genius3).

## 4. Conclusions

A pincer chelating diisatin terephthalyl-dihydrazone ligand (H_2_Lph) was prepared, which was employed for complexation with VO^2+^ and Cu^2+^ ions, as a *bis*-tridentate mono-basic coordinated ligand, within a 1:2 equivalent ratio, respectively. Using the spectroscopic analysis, the structures of the ligand H_2_Lph and its VOLph and CuLph chelated complexes were confirmed.

Biochemically, both VOLph and CuLph chelates were used in a study on their inhibiting effect on the growth of some well-known human cancer cell lines and some common bacteria and fungi, and they demonstrated reported valuable inhibition behavior compared to their free H_2_Lph ligand. Their antiproliferative action against some human cancer cells was examined, displaying high antiproliferative reactivity. Within normal cells (WI-38), the selectivity index was calculated for the studied compounds. The selectivity of the MLphs toward cancer cells was highly appreciable, and hence they may be safer for normal cell populations. VOLph and CuLph complexes demonstrated modified bioaction compared to H_2_Lph (i.e., their un-bonded ligand) regarding their interaction with *ct*DNA, as reported by *K*_b_ (binding constant) and $\Delta G_{b}^{\neq }$ (Gibb’s free energy). On the other hand, VOLph displayed a larger interaction mode with the *ct*DNA solution compared to CuLph based on the binding constants (*K*_b_ = 16.31 and 16.04 × 10^7^ mol^−1^ dm^3^) and on the Gibbs’ free energy values ($\Delta G_{b}^{\neq }$ = −47.11 and −16.04 kJ mol^−1^), respectively. VOLph’s features of lipophilicity (hydrophobicity) and Lewis acidity in addition to ots modified electronegativity could demonstrate the positive role of the VO^2+^ ion (with a high oxidation state) compared to the Cu^2+^ ion (with a low oxidation state) in the interaction of these species with *ct*DNA. All of the above results for the *ct*DNA interactions are supported by the gel electrophoresis and viscosity assays.

## Figures and Tables

**Table 1:** Element percentage analyses of C, H, and N for H_2_Lph, VOLph, and CuLph. The UV/Vis spectrometric titration for their solution in DMF, and their conductivity features (Λ_m_, Ω^−1^ cm^2^ mol^−1^) in DMSO and DMF at 25 °C.

Comp. (M.W.)	Color	CHN Analyses, %			Electronic Spectra			Λ_m_, Ω^−^^1^ cm^2^ mol^−^^1^	
		C	H	N	λ_max_, nm	ε, mol^−1^·cm^−1^	Assign.	DMSO	DMF
H_2_Lph (452.43 g·mol^−1^)	Dark yellow	63.99 (63.71)	3.12 (3.56)	18.31 (18.58)	251	9021	π→π*	25	31
					340	4005	n→π*		
					396	3119	LCT		
VOLph (782.51 g·mol^−1^)	Greenish brown	51.88 (52.19)	3.90 (3.61)	10.29 (10.74)	247	10,185	π→π*	37	41
					296	7922	π→π*		
					313	7031	n→π*		
					389	5204	L-MCT		
					441	4692	*d*→*d*		
CuLph (648.41 g·mol^−1^)	Green	44.82 (44.46)	1.92 (2.18)	12.51 (12.96)	262	8161	π→π*	32	44
					300	6562	n→π*		
					448	4603	*d*→*d*		

**Table 2:** The area of the inhibition zone (mm) for the reactivity of H_2_Lph, VOLph, and CuLph against the studied microbes’ growth at room temperature in DMSO.

Comp.	Zone of Effective Inhibition of the Growth of Three Bacteria (mm)			Zone of Effective Inhibition for the Growth of Three Fungi (mm)		
	*S. marcescens* (−ve)	*E. coli* (−ve)	*S. aureus* (+ve)	*C. albicans*	*A. flavus*	*T. rubrum*
H_2_Lph	19 ± 0.05	18 ± 0.45	19 ± 0.10	17 ± 0.20	13 ± 0.26	16 ± 0.75
VOLph	36 ± 0.55	30 ± 0.45	39 ± 0.65	25 ± 0.80	20 ± 0.85	28 ± 0.55
CuLph	35 ± 0.75	29 ± 0.80	38 ± 0.35	26 ± 0.00	20 ± 0.75	27 ± 0.40
VO(acac)_2_	27 ± 0.35	23 ± 0.75	25 ± 0.25	20 ± 0.65	17 ± 0.45	22 ± 0.50
CuCl_2_·2H_2_O	22 ± 0.20	18 ± 0.10	20 ± 0.05	17 ± 0.70	14 ± 0.15	17 ± 0.35
Gentamycin	40 ± 0.33	37 ± 0.72	46 ± 0.11	--	--	--
Fluconazole	--	--	--	37 ± 0.62	25 ± 0.90	31 ± 0.88

**Table 3:** MIC concentrations for antibacterial and antifungal assays with H_2_Lph, VOLph, and CuLph.

Comp.	MIC (Minimum Inhibition Concentration), μM					
	*S. marcescens* (−ve)	*E. coli* (−ve)	*S. aureus* (+ve)	*C. albicans*	*A. flavus*	*T. rubrum*
H_2_Lph	6.25	6.00	5.80	7.05	6.25	6.75
VOLph	1.50	1.25	1.00	1.25	1.50	1.25
CuLph	1.75	1.50	1.00	1.25	1.75	1.25
DMSO	--	--	--	--	--	--

**Table 4:** Comparison of the inhibition potential of VOLph and CuLph, as dinuclear homoleptic chelates, with other similar hydrazone and dihydrazone complexes.

Comp.	Inhibited Zone (mm) of Bacterial Growth			Inhibited Zone (mm) of Fungal Growth		
	*S. marcescens* (−ve)	*E. coli* (−ve)	*S. aureus* (+ve)	*C. albicans*	*A. flavus*	*T. rubrum*
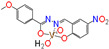 [49]	31 ± 0.99	33 ± 0.05	37 ± 0.12	30 ± 0.52	17 ± 0.07	22 ± 0.83
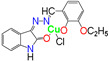 [50]	32 ± 0.19	30 ± 0.29	35 ± 0.05	30 ± 0.77	17 ± 0.82	22 ± 0.35
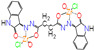 [29]	33 ± 0.75	33 ± 0.42	37 ± 0.85	30 ± 0.90	19 ± 0.75	26 ± 0.05
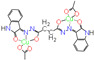 [29]	32 ± 0.10	34 ± 0.00	38 ± 0.05	31 ± 0.52	18 ± 0.35	24 ± 0.60

**Table 5:** The selectivity index for the antiproliferative action of H_2_Lph, VOLph, and CuLph chelators against the human cancer cell lines.

Comp.	Selectivity Index		
	HCT-116	MCF-7	HepG-2
H_2_Lph	1.91	2.98	2.26
VOLph	2.42	3.68	2.42
CuLph	3.01	4.68	3.16
Vinblastine	--	--	--

**Table 6:** The electronic spectra, chromism, ∆*n* changes in absorption bands (λ_max_), the binding constants = *K*_b_, Gibb’s free energy = $\Delta G_{b}^{\neq }$ for the DMSO solutions of H_2_Lph, VOLph, and CuLph with differing concentrations of *ct*DNA solution.

Comp.	λ_free_ (nm)	λ_bound_ (nm)	∆*n*	Chromism		*K*_b_ × 10^7^ mol^−1^ dm^3^	$\Delta G_{b}^{\neq }$ kJ mol^−1^
				%	Type		
H_2_Lph	251	273	22	17.0	Hypo	12.41	−44.05
	340	347	7	6.6			
	399	440	41	36.9			
VOLph	296	270	26	29.1	Hypo	16.31	−47.11
	389	347	42	15.8			
	441	428	13	20.9			
CuLph	262	275	14	22.6	Hypo	16.04	−46.89
	300	311	11	16.3			
	448	453	5	8.1			

## Data Availability

Data are contained within the article and Supplementary Materials.

## Supplementary Materials

The following supporting information can be downloaded at: https://www.mdpi.com/article/10.3390/molecules29020414/s1, Figure S1: ^1^HNMR spectrum of H_2_Lph in DMSO-*d*_6_ at 25 °C; Figure S2: ^13^CNMR spectrum of H_2_Lph in DMSO-*d*_6_ at 25 °C; Figure S3: Continuous variation plot for the stoichiometric molar ratios for VOLph and CuLph complexation formed with H_2_Lph in DMF media at [L] = [VO^2+^] or [Cu^2+^] = 1 × 10^−5^ mol dm^−3^ and 25 °C; Figure S4: pH effect stability of a DMF solution of VOLph and CuLph complexes; Figure S5: EI-Mass spectrum of H_2_Lph in DMF media at 25 °C; Figure S6: EI-Mass spectrum of VOLph in DMF media at 25 °C; Figure S7: The spectral changes for the electronic transitions for H_2_Lph solutions with [DNA] of different *ct*DNA concentrations in DMSO, in μM (at 25 °C); Table S1: Activity index (%) of the antibacterial assay for H_2_Lph, VOLph and CuLph reagents; Table S2: Activity index (%) of the antifungal assay for H_2_Lph, VOLph and CuLph reagents; Table S3: The antiproliferative index of H_2_Lph, VOLph and CuLph reagents versus the current human cancer cell lines.
