# Supplementary material for: Bimetallic bis-Aroyldihydrazone-Isatin Complexes of High O=V(IV) and Low Cu(II) Valent Ions as Effective Biological Reagents for Antimicrobial and Anticancer Assays
Source: Molecules. 2024 Jan 15;29(2):414. doi: 10.3390/molecules29020414 (PMC10820496; doi:10.3390/molecules29020414)
Supplement: Supplementary file 1 [file molecules-29-00414-s001.zip › molecules-2790129-supplementary.pdf]

Supplementary materials

Bimetallic *bis*-Aroyldihydrazone-Isatin Complexes of High O=V(IV) and Low Cu(II) Valent Ions as Effective Biological Reagents for Antimicrobial and Anticancer Assays

Ahmed Khalil [1,2,]* and Mohamed Shaker S. Adam [1,3]

[1]Department of Chemistry, College of Science, King Faisal University, P.O. Box 400, Al-Ahsa 31982, Saudi Arabia
[2]Chemistry Department, Faculty of Science, Zagazig University, Zagazig 44519, Egypt
[3]Department of Chemistry, Faculty of Science, Sohag University, Sohag 82534, Egypt

*Correspondence: akkhalil@kfu.edu.sa

2.1. Devices and methodologies

The essential materials used in the current study (without rehandling) were obtained from various suppliers (BDH, Sigma-Aldrich and Merck). In particular, the main element percentages of C, H and N in H_2Lph, VOLph and CuLph were estimated at an ambient temperature within the V2.3 model of the GMBH varioEI device. For the determination of the melting and/or decomposition points, a Gallenkamp–Sanyo-type apparatus was utilized. Also, at 25 ºC, the molar conducting power of VOLph and CuLph (with concentration = 1.0 × 10_{-3} mol dm_{-3}) was studied in polar medium of *N*,*N*-dimethylformamide (DMF) or dimethyl sulfoxide (DMSO) using a Jenway conductivity meter apparatus. Studying the nuclear magnetic resonance spectroscopy ($_1$H- and $_{13}$C-nuclei) for the ligand H_2Lph in DMSO-*d*6 at 25 °C was achieved using an FT-NMR Bruker multinuclear spectrophotometric device (ARX400 model), in which the field magnetism for $_1$H- and $_{13}$C-nuclei was employed at 400.1 and 100.6 MHz, respectively. At 25 °C, FTIR spectra for H_2Lph, VOLph and CuLph (as solid samples) were studied using a FT-IR spectrophotometer from Agilent Technology (Cary-630 model). The electronic spectrometric titrations for the solutions in DMF with concentrations of 1.0 × 10_{-6} mol dm_{-3} for H_2Lph, VOLph and CuLph were examined at 25 °C using a Shimadzu spectrophotometer (UV-1800 model). MS (m/z) mass spectra for H_2Lph, VOLph and CuLph in DMF were obtained using a mass spectrometric machine (model of Qtof Micro YA263). Magnetic features of the studied complexes were evaluated based on a magnetic susceptibility balance (Sherwood Scientific).

List of Tables

Table S1. Activity index (%) of the antibacterial assay for H_2Lph, VOLph and CuLph reagents.

	Activity index (%)		
Comp.	S. Marcescence (-ve)	E. Coli (-ve)	S. Aureus (+ve)
H_2Lph	47.5	48.7	41.3
VOLph	90.0	81.1	84.8
CuLph	87.5	78.4	82.6

Table S2. Activity index (%) of the antifungal assay for H_2Lph, VOLph and CuLph reagents.

	Activity index (%)		
Comp.	C. albicans	A. flavus	T. rubrum
H_2Lph	45.9	52.0	51.6
VOLph	67.6	80.0	90.3
CuLph	70.3	80.0	87.1

Table S3. IC_{50}, the antiproliferative index of H_2Lph, VOLph and CuLph reagents versus the current human cancer cell lines.

	IC_{50} (μM)			
Comp.	HCT-116	MCF-7	HepG-2	WI-38
H_2Lph	67.56±0.12	43.00±0.55	56.75±0.50	128.15±0.25
VOLph	38.30±0.72	25.10±0.36	38.05±0.25	92.70±0.90
CuLph	39.09±0.64	25.25±0.45	37.80±0.45	117.36±0.33
Vinblastine	13.30±0.11	4.12±0.14	7.50±0.10	--

List of Figures

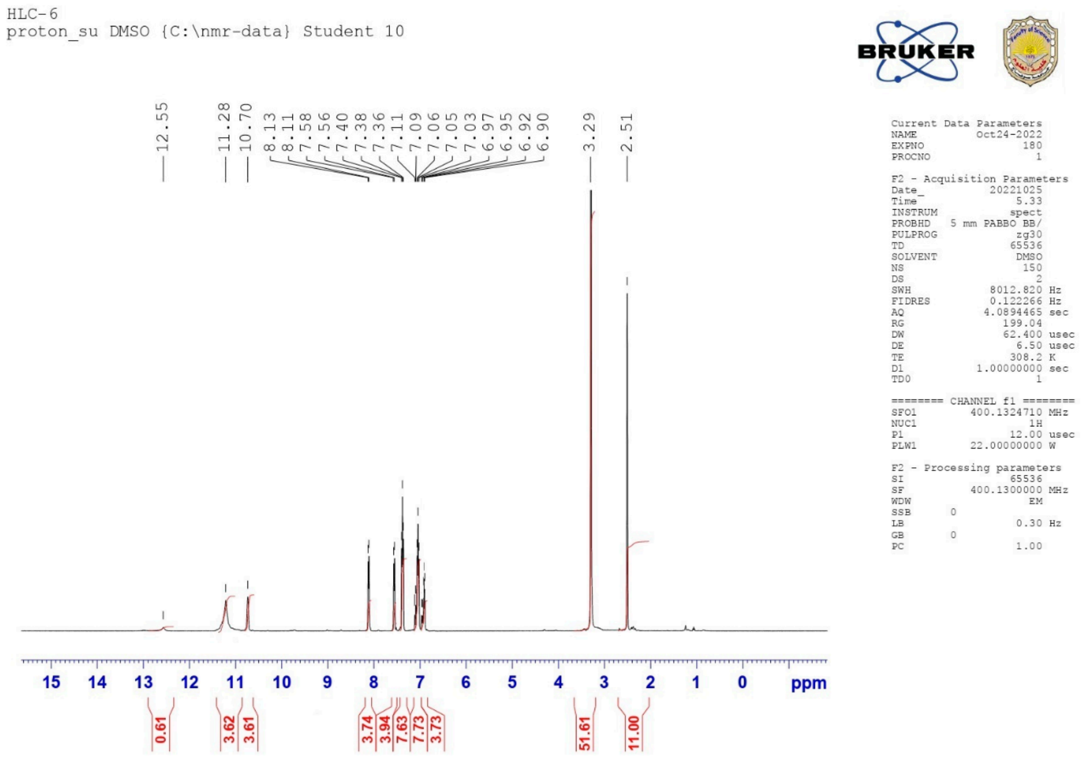

Figure S1. ^{1}HNMR spectrum of H_2Lph in DMSO-d_6 at 25 °C.

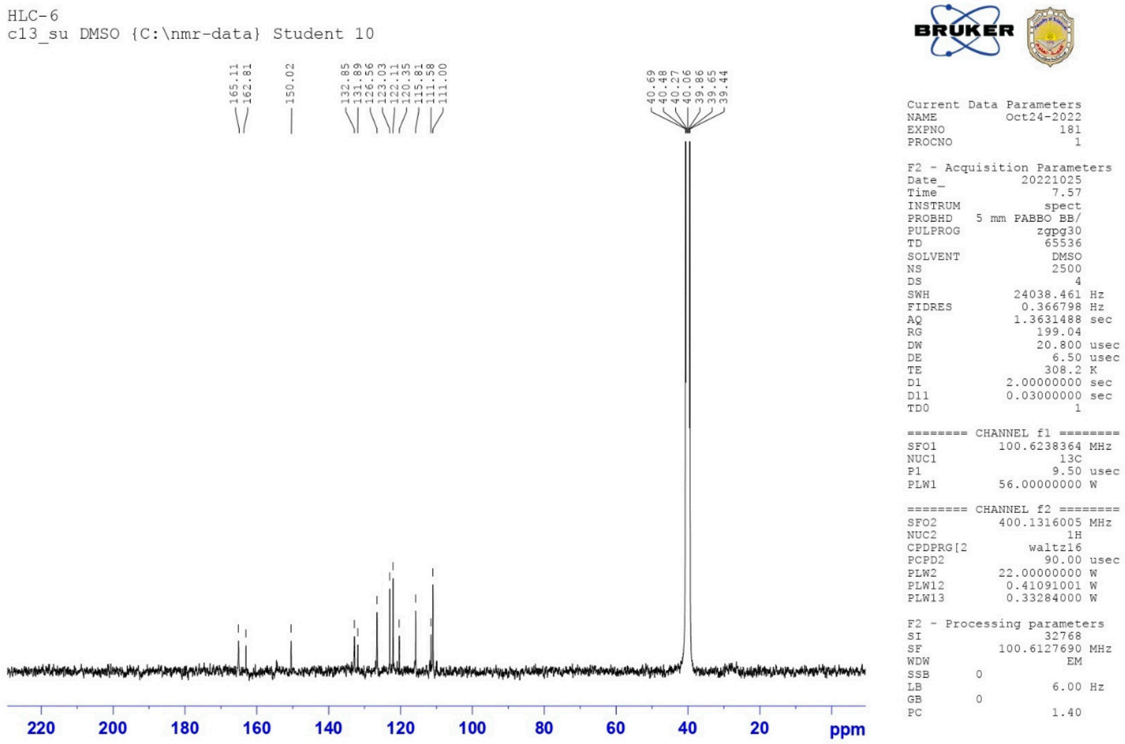

Figure S2. ^{13}CNMR spectrum of H_2Lph in DMSO-d_6 at 25 °C.

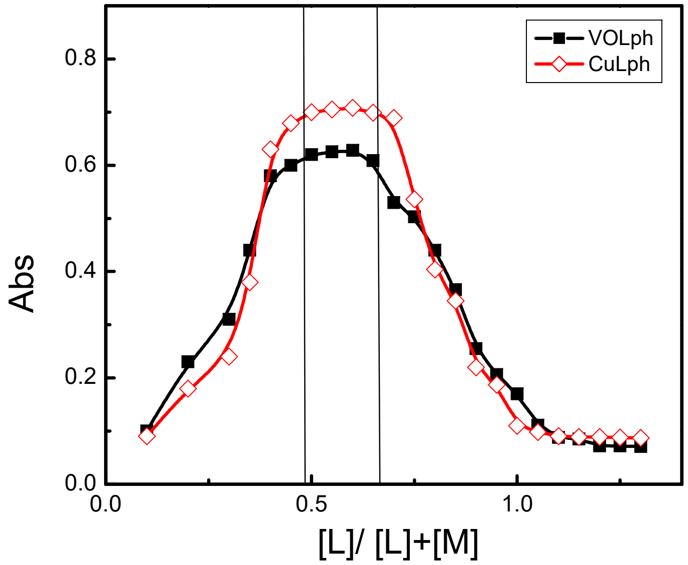

Figure S3. Continuous variation plot for the stoichiometric molar ratios for VOLph and CuLph complexation formed with H_2Lph in DMF media at [L] = $[VO^{2+}]$ or $[Cu^{2+}]$ = 1×10^{-5} mol dm^{-3} and 25 °C.

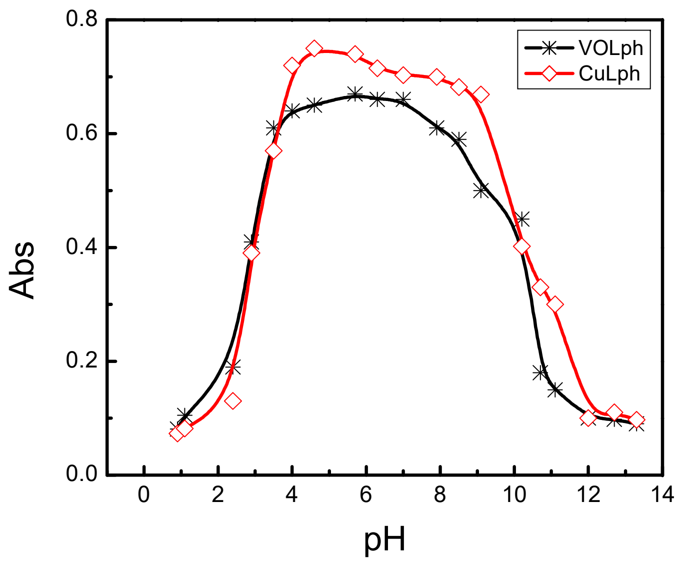

Figure S4. pH effect stability of an DMF solution of VOLph and CuLph complexes.

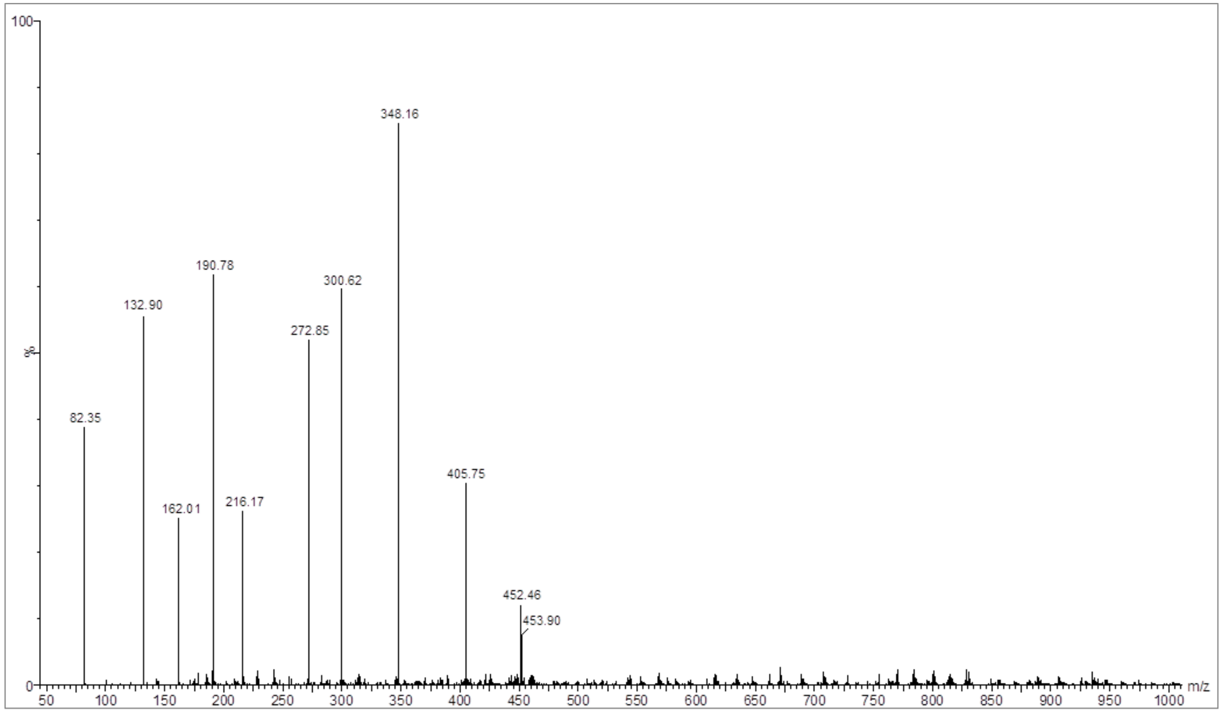

Figure S5. EI-Mass spectrum of H_2Lph in DMF media at 25 °C.

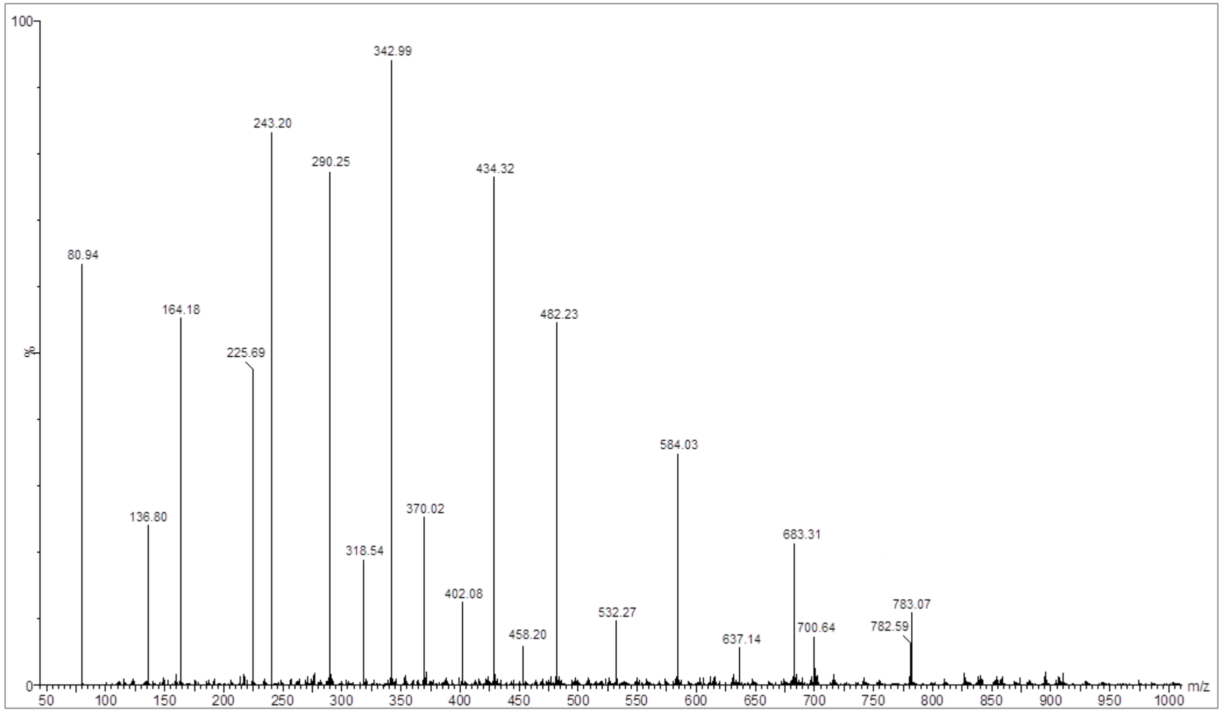

Figure S6. EI-Mass spectrum of VOLph in DMF media at 25 °C.

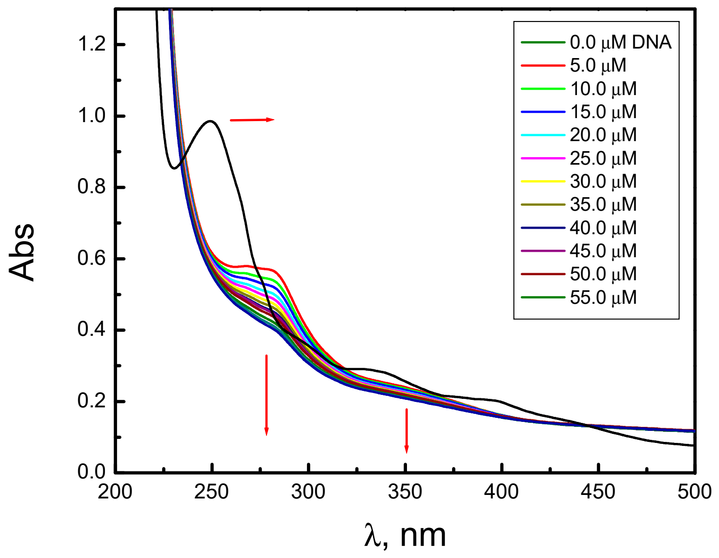

Figure S7. The spectral changes for the electronic transitions for H_2Lph solutions with [DNA] of different *ct*DNA concentrations in DMSO, in μM (at 25 °C).
